# Management of a large abdominal dermatofibrosarcoma protuberans requiring a life-threatening excision: A case report

**DOI:** 10.1016/j.ijscr.2025.111579

**Published:** 2025-06-27

**Authors:** Rawan Albadia, Perrine Rousset, Damien Massalou, Olivier Camuzard, Henri Montaudié, Elise Lupon

**Affiliations:** a University Institute of Locomotor and Sport (IULS), Pasteur Hospital, Nice, France; b Dermatology Department, Université Côte d'Azur, France; c Digestive Surgery Unit, University Hospital of Nice, Université Côte d'Azur, France; d INSERM U1065, Centre Méditerranéen de Médecine Moléculaire, Université Côte d'Azur, Nice, France; e Laboratory of Molecular PhysioMedicine (LP2M), UMR 7370, CNRS, University Côte d'Azur, Nice, France

**Keywords:** Dermatofibrosarcoma protuberans, DFSP, Imatinib, Perforators flaps

## Abstract

**Introduction and importance:**

Dermatofibrosarcoma protuberans (DFSP) is a rare malignant tumor of the dermis and subcutaneous tissue, characterized by local aggressiveness and a high recurrence rate. The gold standard treatment is wide excision with negative margins, sometimes using Mohs surgery, with careful planning for reconstruction. In challenging cases, neoadjuvant imatinib therapy and adjuvant radiotherapy may help optimize outcomes. We report a case of extensive abdominal DFSP requiring a multidisciplinary approach after resection exposed the liver and the last three right ribs.

**Case presentation:**

Neoadjuvant imatinib was administered to reduce tumor size, followed by radical resection and immediate reconstruction using prosthetic mesh and three pedicled flaps: a deep inferior epigastric perforator flap, an anterior intercostal artery perforator flap, and a pedicled latissimus dorsi flap. The reconstruction was performed as a single-stage procedure. Postoperative ischemia due to hematoma required conversion of the latissimus dorsi flap into a free flap and remobilization of the remaining flap. A split-thickness skin graft was applied on postoperative day ten. Histology confirmed negative margins. At six months, the patient showed complete healing, no recurrence, and a satisfactory reconstructive outcome.

**Clinical discussion:**

In such extensive DFSP cases, immediate flap reconstruction helps prevent complications related to exposed bone or viscera and preserves functional and aesthetic outcomes. It does not hinder oncologic follow-up and may reduce morbidity.

**Conclusion:**

This case highlights the surgical and reconstructive challenges of large DFSPs and the vital role of a plastic surgery team in planning tailored, multidisciplinary management within an oncodermatology center.

## Highlights

• Neoadjuvant imatinib can significantly reduce DFSP - tumor size before surgery.
• Large abdominal DFSP excision may expose vital structures requiring complex reconstruction.
• Immediate flap and mesh reconstruction ensures functional and aesthetic outcomes.
• A multidisciplinary team is crucial for managing extensive DFSP defects safely.

## Graphical abstract

**Figure:**
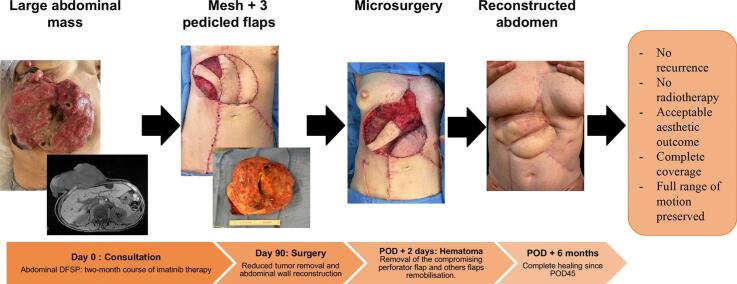

## 1 Introduction and importance

Dermatofibrosarcoma protuberans (DFSP), also known as Darier-Ferrand tumor, was initially characterized by Darier and Ferrand in 1924 [1]. It is a rare and slow-growing malignant soft tissue neoplasm originating from the dermis and subcutaneous tissues. Although it exhibits a low propensity of distant metastasis, its highly infiltrative nature aggravates local recurrences in up to 60 % of patients [2]. The precise etiology of DFSP remains unclear; however, it is strongly associated with chromosomal translocation between chromosomes 17 and 22, leading to platelet-derived growth factor gene alterations. This tumor primarily affects adults aged 30–50 years and is more prevalent among women and individuals of African descent [[3], [4], [5]]. A definitive diagnosis is established by excisional or punch biopsy, accompanied by immunohistochemical staining.

Histologically, DFSP is classically characterized by monomorphic spindle cells arranged in a storiform pattern. Several histologic variants exist, including myxoid, pigmented, myoid, granular cell, sclerosing, atrophic, and fibrosarcomatous subtypes, with the latter being the only variant manifesting distinct clinical behavior [6]. Immunohistochemically, DFSP demonstrates strong, diffuse positivity for CD34, a monomeric 115 kDa glycoprotein expressed on normal hematopoietic progenitor cells. Additional positive markers include apolipoprotein D and nestin [1]. Management depends on many factors, considering the radical excision with clear surgical margins to be the gold standard choice [5].

While various aspects of DFSP have been well documented—including clinical presentation, recommended resection margins, and the role of adjuvant therapy—the reconstructive strategies have received comparatively less attention. Yet, despite the reduction in required margins made possible by advances in histopathological analysis [7], resections can remain extensive and technically challenging in cases of large tumors, requiring dedicated reconstructive expertise [8].

We present the case of a woman with an extensive abdominal DFSP which, following resection, resulted in exposure of the liver and the last three right true ribs, requiring multidisciplinary management and a customized reconstructive approach.

This case report has been reported in line with the SCARE checklist [9].

## 2 Case presentation

A 66-year-old Caucasian women, Fitzpatrick phototype II skin, was referred to the dermatology department with a chief complaint of a nodular mass that was progressively enlarging and ulcerating, located on the right hypochondriac and lumbar regions, and extending into the umbilical area. The patient reported a gradual increase in the lesion over three years but had not pursued medical care. Furthermore, she showed overall health deterioration with a significant weight loss of 15 kg. Her medical history was unremarkable, except for a cesarean section. She lived alone, employed as a cleaner, and smoked one pack daily while occasionally consuming alcohol.

**Fig. 1:**
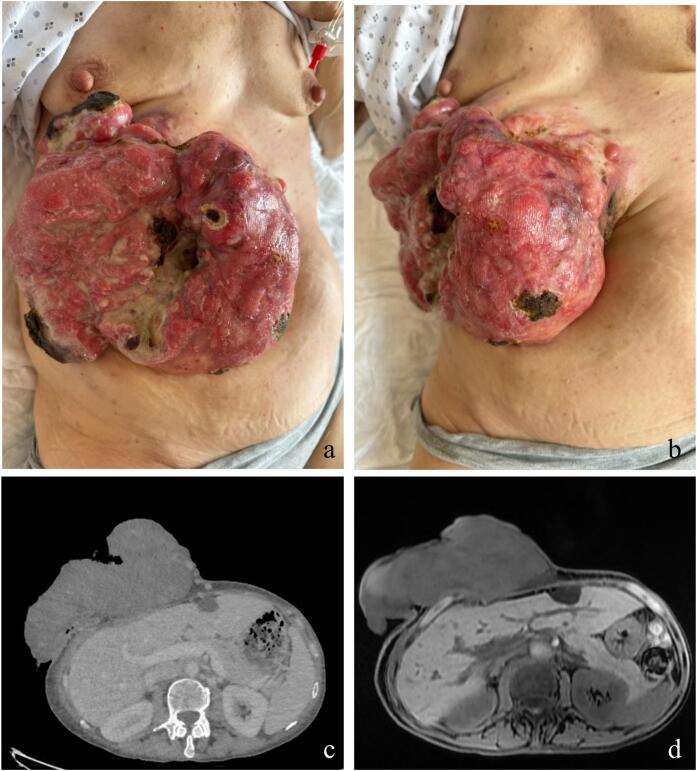
Pre-operative considerations of the large abdominal DFSP. a. Top view photograph of the voluminous abdominal mass. b. Photograph of the ulcerated nodular abdominal mass, showing the tumor base located along the midline. c. CT scan showing a lesion invading the upper portion of the rectus abdominis muscle and hepatic scalloping at the junction of segments IV and V, two months prior to surgery. d. MRI performed two months prior to surgery, showing a tumor measuring 20 cm in transverse diameter, 11 cm in maximal depth, and 19 cm in vertical height on the selected view. The mass was in close contact with the liver and the right 6th to 10th chondrosternal cartilages, without clear evidence of invasion.

During physical examination, a voluminous ulcerative mass measuring 25 × 30 cm was observed, displaying exudation, fibrinous deposits, and necrotic tissue (Fig. 1a and b). Cardiac auscultation revealed a systolic murmur in the aortic region. Laboratory results indicated microcytic anemia (haemoglobin: 5.1 g/dL) for which she required blood transfusions, an inflammatory response characterized by elevated leukocytes (11.84 G/L) and C-reactive protein (CRP: 167 mg/L). CT scan of the chest, abdomen and pelvis revealed a large soft-tissue mass originating from the subcutaneous tissues of the anterior abdominal wall, predominantly on the right side. The lesion appeared to invade the upper portion of the rectus abdominis muscle and exhibited hepatic scalloping at the junction of segments IV and V. (Fig. 1.c). MRI further characterized the lesion as a large, protruding mass of the right anterior abdominal wall, with no evidence of intraperitoneal or hepatic involvement (Fig. 1.d). The lesion was in contact with the distal portions of the last three true ribs on the right but showed no evidence of pleural or diaphragmatic invasion. Excisional biopsy of the mass revealed a spindle cell proliferation with diffuse CD34 positivity. Ki-67 immunostaining demonstrated heterogeneous nuclear positivity in less than 1 % of cells, consistent with a diagnosis of DFSP. Molecular studies confirmed the presence of the COL1A1-PDGFB fusion gene.

**Fig. 2:**
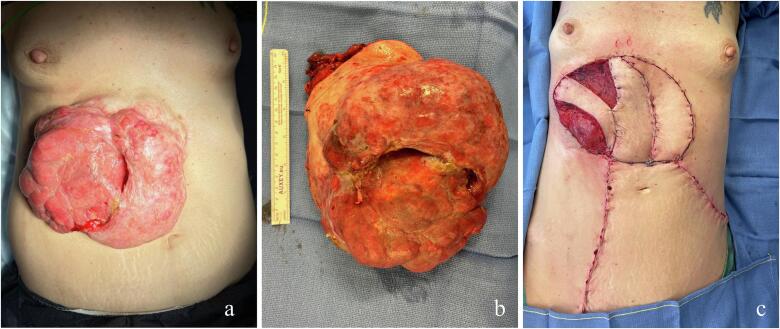
Per-operative considerations of the large abdominal DFSP. **a**. Intraoperative view of the tumor on the day of resection, showing a good response to a two-month course of imatinib therapy. b. Tumor resected on the operative table. c. Immediate post-operative photographs showing the three pedicled flaps. Left: pedicled latissimus dorsi (LD) flap with skin paddle; center: deep inferior epigastric perforator (DIEP) flap; right, along the midline: anterior intercostal artery perforator (AICAP) flap.

A multidisciplinary tumor board discussed the advantages of a single-stage procedure with wide 3 cm margins versus a two-stage surgical approach consisting of radical excision followed by delayed abdominal wall reconstruction, based on slow Mohs micrographic analysis. Given the patient's profile, anticipated exposure of critical anatomical structures and the minimal functional impact of removing 3 cm margins relative to the extensive size of the tumor, the team opted for a single-stage procedure. This was to be preceded by a two-month course of neoadjuvant targeted therapy with imatinib (600 mg/day) to reduce tumor volume. Surgical excision was performed three months after the completion of targeted therapy with imatinib 600 mg/day, following a good tolerance and a favourable response evidenced by a 40 % reduction in tumor size (Fig. 2.a).

Three months after the initial consultation, the procedure was performed jointly by the reconstructive and general surgery teams. It proceeded as planned with a wide local excision, ensuring 3 cm lateral margins and achieving deep margins at the level of the liver, while preserving both the costal periosteum and the deep peritoneum. Given the infectious risk associated with this surgery, perioperative antibiotic prophylaxis was administered by the infectious team. The resection resulted in exposure of the liver and the last three true ribs due to tumor adherence to these structures, without any macroscopic invasion. The oriented surgical specimen was sent to the pathology department for standard analysis. (Fig. 2.b). The resulting defect was circular and measured 14 × 25 cm. This extensive defect necessitated immediate reconstruction within the same intervention to avoid possible complications if left for a second-step procedure, and without waiting for confirmation of margin status by frozen section. Abdominal wall reconstruction was performed in two stages during the procedure. First, a Phasix ST mesh (Becton, Dickinson and Company, Franklin Lakes, NJ, USA) was placed by the gastrointestinal surgery team as a pre-peritoneal prosthesis, anchored to the posterior sheath, the inferior aspect of the linea alba, the costal periosteum laterally, and the xiphoid process superiorly. The mesh extended at least 3 cm beyond the defect in all directions, except for a small, uncovered area approximately 1 cm to the left of the xiphoid. Second, soft tissue reconstruction was done out by the plastic surgery team using a combination of pedicled Deep Inferior Epigastric Perforator (DIEP) and Anterior Intercostal Artery Perforator (AICAP) flaps. Additionally, for achieving complete coverage of the defect, a pedicled latissimus dorsi flap (pLD) with a skin paddle for postoperative monitoring was done. Closed-suction drainage was routinely placed at the latissimus dorsi donor site to prevent fluid collection and promote optimal healing. These pedicled flaps preserved vascular integrity at the end of the intervention (Fig. 2.c).

On postoperative day two, the patient developed ischemia of the AICAP flap. Surgical reintervention was undertaken to remove the compromised perforator flap, whose pedicle had been avulsed by a hematoma. The DIEP flap also showed signs of venous congestion, with pedicle compression caused by the hematoma. After hematoma evacuation, the pLD flap was converted into a free flap to achieve adequate coverage and was microsurgically anastomosed to the left internal mammary vessels. Both flaps demonstrated good viability, with residual signs of venous congestion in the DIEP flap, which improved after hematoma evacuation (Supplemental Fig. 1). At day 10, a 0.3 mm thick, 2:1 meshed split-thickness skin graft was applied to the muscular portion of the reconstruction, taken from the inner aspect of the thigh. As intraoperative microbiological samples returned positive during this surgery, the patient was subsequently treated with dual antibiotic therapy, a course of antibiotics of Ceftazidime IV for 10 days and Ciprofloxacin for 3 weeks was completed.

**Fig. 3:**
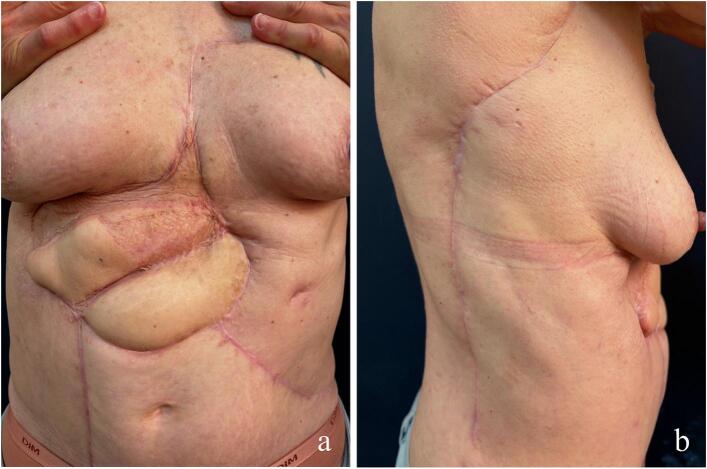
Post-operative photograph at 6 months showing complete cicatrisation of the flaps and skin graft. **a.** Frontal view showing complete healing of the flaps and skin grafts over the muscular portion of the latissimus dorsi flap. **b.** Lateral view showing the donor site scar from the latissimus dorsi flap harvest.

She showed good response and well vascularization afterward as well as no further major complication noted. Complete healing was achieved by day 45. Histopathological examination of the resected abdominal mass confirmed DFSP, with infiltration into the subcutaneous tissue and focal involvement of the superficial fascia. Lateral margins were free of tumor, with the closest measuring 0.4 and 0.9 cm, while deep margins were also negative. The lesion was classified as FNCLCC Grade 1, indicating low-grade malignancy, and did not require adjuvant radiotherapy. At the four-month postoperative follow-up visit with the dermatology team, the patient was in stable condition and reported no significant complaints, except for occasional lymphatic fluid discharge from the scarred area on the lower right flank which was completely resolved within the next month. MRI conducted at the time of this visit and demonstrated post-therapeutic changes in the anterior abdominal wall, with no evidence of suspicious lesions. At the six-month postoperative follow-up visit with the plastic surgery team, she showed no signs of recurrence, no abdominal hernia, and had no limitations in daily activities (Fig. 3).

Overall, the patient demonstrated a favourable therapeutic response without significant complications. Annual oncological follow-up will allow monitoring for the absence of recurrence.

## 3 Clinical discussion

The management of DFSP is influenced by factors such as tumor size, location (head, neck, genitalia, hands, feet, etc.), and the potential impact on functional or cosmetic abilities. Imatinib, a drug that inhibits tyrosine kinase, is employed as neoadjuvant therapy in specific cases where surgical resection poses significant challenges. Based on the literature review [[10], [11], [12]], daily doses of 400 and 800 mg have been found to have an equal impact on reducing tumor size, and lower doses are associated with fewer side effects [13]. Radiotherapy can be integrated as adjuvant therapy in cases of recurrence or when negative clear margins cannot be achieved [2,5,13,14]. Gold standard treatment is a complete surgical resection, typically achieved by negative surgical margins (2–3 cm) of either wide local excision (WLE) or Mohs micrographic surgery [5]. As mentioned, the patient benefited from a two-month course of Imatinib, which led to significant tumor shrinkage and underwent WLE with lateral surgical margins of 3 cm without awaiting intraoperative histopathological confirmation via frozen section.

In certain instances, as clearly as in this case, these tumors can lead to severe functional impairments in exposed bone or organs, requiring a complex flap reconstruction procedure. Performing immediate flap reconstruction during surgical excision in such critical cases is essential to minimize the risk of deep infections and other complications [15]. Moreover, primary flap reconstruction ensures both functional and aesthetic benefits without compromising the ability to detect disease recurrence. This approach supports complete removal of the tumor, preserves patient function, and enhances survival rates. It is crucial to note that the need for advanced reconstructive techniques should not limit the extent of surgical margins or compromise the thorough removal of the tumor [15,16]. Due to the high risk of abdominal wall hernia and infection associated with leaving the abdominal wall exposed pending a secondary intervention, a biosynthetic Phasix ST mesh (Becton, Dickinson and Company, Franklin Lakes, NJ, USA) was placed in the retromuscular position as a strategic solution for immediate abdominal wall coverage. Timely management of the open abdomen is critical, as inadequate coverage may result in progressive visceral edema, compromised perfusion, and potential multi-organ dysfunction. Also, prolonged exposure of intra-abdominal contents increases the likelihood of infection and adhesion formation, thereby complicating subsequent definitive closure [17,18]. Modern multidisciplinary strategies aim to achieve early single-stage closure of open abdomen, using tailored techniques based on defect size and patient condition [19]. Flap-based reconstruction was essential, as skin grafting was contraindicated due to the tumor's removal significant exposition of the liver and bony structures, creating a complex defect that necessitated immediate, durable and reliable soft tissue coverage. Flaps offer enhanced vascularization, mechanical compatibility with soft tissues, and the ability to effectively cover exposed bone and cartilage [20]. That serves in cases requiring adjuvant radiotherapy when needed afterward and is well-documented in the literature that the flaps reduce the risk of radiation-induced necrosis [21]. Additionally, in a retrospective series of 25 cases published in 2025, the use of flaps for reconstruction of DFSP defects was associated with improved aesthetic and functional outcomes [8].

In our case, the reconstruction approach was performed initially using a combination of pedicled flaps of DIEP, AICAP alongside a pLD flaps. The DIEP flap, a perforator flap based on the deep inferior epigastric artery, is widely recognized for its efficacy in breast and abdominal wall reconstruction with most literature focused on its use. However, its versatility extends to reconstructions in the head and neck, extremities, gynaecological, and genitourinary regions. Although predominantly employed as a free flap, there are reports documenting its use in a pedicled application [22]. The AICAP flap is a regional perforator flap selected for abdominal wall reconstruction due to its consistent vascular supply from anterior intercostal artery perforators and ensuring reliable perfusion. Its design permits for transposition or V—Y advancement, making it adaptable for various thoracic and abdominal defects [23]. The pLD flap, based on the thoracodorsal artery, has proven to be a reliable option for abdominal wall defect reconstruction. It offers durable coverage and excellent aesthetic and functional outcomes, particularly in complex cases [24].

The initial use of perforator flaps was chosen to avoid the risks associated with microvascular surgery and to minimize additional incisions; however, it was not the only flap-based reconstruction option available. In our case, the patient's history of heavy smoking was taken into account when selecting pedicled perforator flaps over free flaps, as smoking is known to increase the risks of flap failure, hematoma formation, and fat necrosis [25]. When a hematoma compromised the viability of the AICAP flap, the flap was removed and the pLD flap was converted into a free flap. This microsurgical conversion was necessary to gain additional coverage surface by facilitating mobilization of the muscle flap, which was not possible in its pedicled form. The free flap alone was insufficient to cover the entire defect, and the DIEP perforator flap was still required to reconstruct the left side of the defect. Additionally, a split-thickness skin graft was applied to the muscular portion of the reconstruction to complete the coverage once flaps viability was confirmed at day 10.

In cases of extensive resection as in our case, the risk of complications including hematoma formation and flap viability issues is significant, requiring very close monitoring—hourly in our practice—of the flaps during the first 72 h, especially. The reconstructive team must be trained in the monitoring of perforator flaps, as signs of venous congestion are reversible if hematoma evacuation is performed promptly. This is illustrated by the evolution of venous congestion signs (see Supplemental Fig. 1), which had fully resolved in the postoperative period (Fig. 3). Given the level of expertise required in such cases, collaboration between the dermatology and reconstructive surgery teams proves to be essential in such cases. However, this multidisciplinary partnership remains insufficiently developed in many center [26].

This report, following the SCARE guidelines [9] highlights the complex surgical management of an advanced DFSP lesion. A multidisciplinary approach integrating targeted therapy, radical excision, and reconstructive surgery ensured optimal oncologic and functional outcomes.

## 4 Conclusion

This case of a large DFSP illustrates the complex reconstructive challenges posed by an unusually voluminous tumor. The successful excision and reconstruction highlight the importance of a multidisciplinary surgical approach and the essential role of plastic surgery within an oncodermatology center. Through coordinated efforts across specialties, complete resection with clear margins was achieved, postoperative complications were effectively managed, and acceptable soft tissue coverage was provided.

The following are the supplementary data related to this article.

Supplemental Fig. 1 Intraoperative view at day 2 during surgical revision for hematoma. The latissimus dorsi flap was remobilized using its microvascular anastomoses to cover the defect following removal of the AICAP flap. The purplish appearance of the DIEP skin paddle reflects venous congestion due to pedicle compression by the hematoma. Supplemental Fig. 1

## Consent

Written informed patient consent was gained for the submission of this case report.

## Ethics statement

The authors have nothing to report.

## Guarantor

All authors in the article accept full responsibility for the work, have access to the patient's information, and decide to publish.

## Author contributions

Rawan Albadia: formal analysis, data curation, writing – original draft, writing – review and editing. Olivier Camuzard: conceptualization, data curation, writing – review and editing. Perrine Rousset, Damien Massalou and Henri Montaudié: conceptualization, data curation, methodology, writing – review and editing. Elise Lupon: conceptualization, data curation, investigation, methodology, formal analysis, project administration, writing – review and editing.

## Sources of funding

None.

## Declaration of competing interest

The authors declare no conflicts of interest.
