# Supplementary figures and images for: Management of a large abdominal dermatofibrosarcoma protuberans requiring a life-threatening excision: A case report
Source: Int J Surg Case Rep. 2025 Jun 27;133:111579. doi: 10.1016/j.ijscr.2025.111579 (PMC12266473; doi:10.1016/j.ijscr.2025.111579)

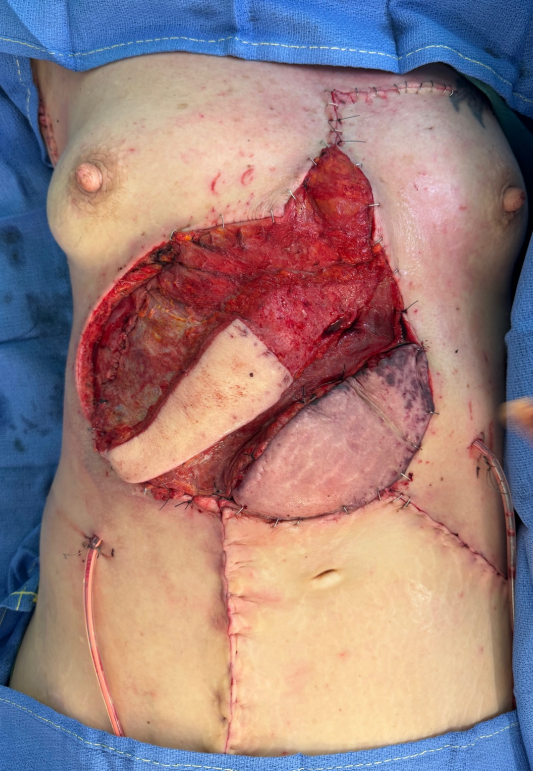

Supplement: Supplemental Fig. 1 — Intraoperative view at day 2 during surgical revision for hematoma. The latissimus dorsi flap was remobilized using its microvascular anastomoses to cover the defect following removal of the AICAP flap. The purplish appearance of the DIEP skin paddle reflects venous congestion due to pedicle compression by the hematoma. [file mmc1.pdf]
